# Coconut Palm: Food, Feed, and Nutraceutical Properties

**DOI:** 10.3390/ani12162107

**Published:** 2022-08-17

**Authors:** Khairiyah Mat, Zulhisyam Abdul Kari, Nor Dini Rusli, Hasnita Che Harun, Lee Seong Wei, Mohammad Mijanur Rahman, Hazreen Nita Mohd Khalid, Muhamad Hakim Mohd Ali Hanafiah, Suniza Anis Mohamad Sukri, Raja Ili Airina Raja Khalif, Zamzahaila Mohd Zin, Mohamad Khairi Mohd Zainol, Mira Panadi, Mohamad Faiz Mohd Nor, Khang Wen Goh

**Affiliations:** 1 Faculty of Agro Based Industry, Jeli Campus, Universiti Malaysia Kelantan, Jeli 17600, Kelantan, Malaysia; 2 Institute of Food Security and Sustainable Agriculture, Jeli Campus, Universiti Malaysia Kelantan, Jeli 17600, Kelantan, Malaysia; 3 Faculty of Fisheries and Food Science, Universiti Malaysia Terengganu, Mengabang Telipot, Kuala Nerus 21030, Terengganu, Malaysia; 4 Department of Clinical Sciences and Sport Technology, School of Biomedical Engineering and Health Sciences, Universiti Teknologi Malaysia, Jalan Pontian Lama, Skudai, Johor Bahru 81300, Johor, Malaysia; 5 Faculty of Data Science and Information Technology, INTI International University, Nilai 71800, Negeri Sembilan, Malaysia

**Keywords:** coconut, alternative feed, nutrition, livestock sectors, functional properties

## Abstract

**Simple Summary:**

Different components of the coconut are being looked into and used as a potential substitute to create or substitute animal feed components. Different coconut products and by-products—such as coconut water, milk, copra, testa, flour, raw kernels, oil, and desiccated coconut—are used with livestock, especially ruminants and aquaculture industries. However, the use of coconut in animal feed may be limited by several things that make it less nutritious. There is a possibility to research new technologies, such as pre-treating coconut to reduce the effects of anti-nutritional substances before they can be used to feed the animals. This review article describes a few important discoveries, which gives a somewhat hopeful view of the future. Different parts of the coconut can and should be used more in animal feed. Coconut in animal feed makes it much cheaper to feed animals and helps them in the digestion process, growth, and health. However, innovative methods of processing, extracting, and treating coconut need to be encouraged to improve nutritional quality and make coconut products function efficiently in feed.

**Abstract:**

The price of traditional sources of nutrients used in animal feed rations is increasing steeply in developed countries due to their scarcity, high demand from humans for the same food items, and expensive costs of raw materials. Thus, one of the alternative sources is coconut parts or coconut as a whole fruit. Coconut is known as the ‘tree of abundance’, ‘tree of heaven’, and ‘tree of life’ owing to its numerous uses, becoming a very important tree in tropical areas for its provision of food, employment, and business opportunities to millions of people. Coconut contains a rich profile of macro and micronutrients that vary depending on the parts and how they are used. It is frequently chosen as an alternative source of protein and fiber. Its uses as an antibacterial agent, immunomodulant, and antioxidant further increase its importance. Using coconut oil in ruminant feed helps to minimize methane gas emissions by 18–30%, and to reduce dry matter intake up to 4.2 kg/d. The aquaculture sectors also use coconut palm as an alternative source because it significantly improves the digestion, growth, lipid metabolism, health, and antioxidative responses. However, coconut is not widely used in poultry diets although it has adequate amount of protein and carbohydrate due to anti-nutritional factors such cellulose (13%), galactomannan (61%), and mannan (26%). This review considered the importance and potential of coconut usage as an alternative ingredient in feed and supplements in various livestock sectors as it has plentiful nutrients and functional qualities, simultaneously leading to reduced feed cost and enhanced production.

## 1. Introduction

*Cocos nucifera*, or coconut palm, is a tall tree grouped with the Palmae or Arecaceae family of monocotyledons. As one of the world’s 10 most beneficial trees, coconut is known as the tree of abundance, heaven, and life for its numerous uses in cosmetics, medicine, and nutrition [1]. According to Karandeep et al. [2], coconut is a very important tree in tropical areas since it provides food, employment, and business opportunities to millions of people. Because of its intrinsic rich macro and micronutrient profile for human health and nutrition, the fruit is known as ‘wonder fruit’. It also naturally disperses well since the fruits can still germinate on land after floating in sea water for up to 120 days, enabling a wide distribution of the tree globally. This dispersal characteristic allows this species to spread far from its origins without the need for human intervention, and to grow into dense monospecific thickets [3]. There are two types of coconuts: dwarf and tall. The time for a variety to bear fruit depends on the variety, with the dwarf varieties grow quickly and fruit after 4 to 5 years of planting, whereas the tall types grow slowly and fruit after 6 to 10 years [4]. The world’s major coconut producers—Indonesia, The Philippines, and India—produce 61, 18, and 15.85 million tonnes annually, respectively [5].

Examples of biotechnological intervention in coconut plant farming include in vitro propagation, genetic improvement of strains, coconut plant irrigation, and rhizophore control. A well-considered cultivation strategy should allow for increased quality of breed and output. The agro-processing of coconut goods is also vital to improve of the plant’s fruits. Local communities would benefit from having a good understanding of the coconut plant and its products [6]. Coconut water or coconut juice mainly consists of minerals, sugars, and low matter content (2–5% wet basis). However, its characteristics do not solely depend on sugars and minerals; it also has unique flavors and qualities. Limited studies have conclusively determined the components that contribute to different characteristics of its quality [7]. Because of its high fat content, the kernel or coconut pulp has been providing monetary benefits for more than a century, and coconut is beyond providing only oil seed. Copra, often known as dried kernel, is a valuable worldwide product. The copra-derived lauric oil is processed into detergent or margarine by the chemical and food industries, respectively. The market for fresh coconut has grown by 300 percent within 20 years, showing coconut as both a global oil commodity and a lucrative fresh fruit [8].

Coconut proteins, oil, and water are all important in addition to derived products such as coconut palm sugar (a value-added product), inflorescence, haustorium, and several nutraceuticals. These dietary supplements can help to avoid nutrient deficiencies and provide health benefits. Aside from cooking oil, coconut oil has great medical values, and it can be used for skin care, both cosmetically and therapeutically. Coconut is regarded as a good source of holistic nutrients in modern times to help with malnutrition and sickness. Coconut oil provides medium-chain triglycerides and lauric acid; both components contain valuable nutritional and medicinal properties [6]. The coconut industry’s most economically impactful product is the virgin coconut oil (VCO). The oil is extracted mechanically with or without heat from a fresh, mature coconut kernel. VCO extraction avoids refining, bleaching, or deodorization in order to preserve its natural qualities. The extraction leaves a white residue or meal known as ‘virgin coconut meal’ (VCM). The lauric acid and saturated fatty acids found in VCO have been shown to have antibacterial properties [9]. They have potential antibiotic components to inhibit infections in ruminants, including sheep and goats, against *Staphylacoccus aureus* that causes subclinical mastitis or mastitis. Coconut flour production has lately expanded due to its useful features, which include the prevention of ailments such as diabetes, colon cancer, and cardiovascular disease [10]. Fruits and vegetables are crucial health foods because they contain a variety of nutrients—including antioxidants, vitamins, fiber, minerals, and sugars—as well as being thirst-quenching and refreshing. Waste produced by the nut, vegetable, and fruit industries is rich in nutrients; it should be repurposed as value-added food products, resulting in significant economic gains [11,12].

Therefore, this review explores the opportunities and potential of applying coconut in the animal feed sector. The composition and functional qualities of coconut; its antibacterial, immunomodulatory, and antioxidative capabilities; and the effects of its inclusion in animal feed for ruminants, poultry, and aquatic animals are the subject of this paper. The coconut’s future prospects will also be discussed.

## 2. Composition of Coconut

**Figure 1:**
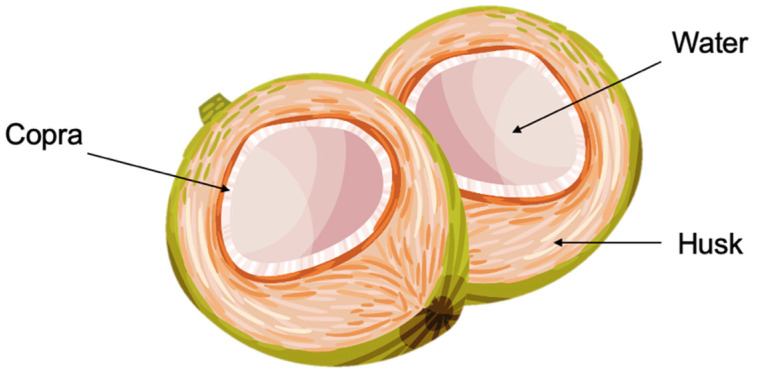
Parts of the coconut.

Coconut that grows on the coconut palm is consumed worldwide, particularly in Southeast Asia and the Caribbean. Its composition is determined by several factors, such as age and variety. Every month, a new cluster of coconuts grows from the previous one. Over the course of a year, both the weight of the kernel and volume composition of the coconut water alter significantly as the coconuts expand in size. The composition of different coconut parts (Figure 1) before and after different processing methods is listed in Table 1.

Coconut water is used in a variety of ways and considered to be one of the functional natural products available. Because it is nutritious and healthful, this pleasant beverage is consumed all around the world. Recently, there has been growing scientific support regarding the importance of coconut water in therapeutic applications and health. Its vast range of applications can be attributed to its chemical composition, which includes carbohydrates, phytohormones, amino acids, minerals, and vitamins [13]. The (+)-catechin was 0.344 g/mL, and (−)-epicatechin was 0.242 g/mL in coconut water [28]. Additionally, as a nutrient-dense medium, coconut milk; fluids produced from pressing copra of matured coconuts promotes the growth of common harmful bacteria. The content of the mesocarp itself influences the content of coconut milk [29]. The variation in oil content and the yield of coconut milk directly relates to the mesocarp’s age [30]. Apart from the coconut’s age and variety, the content of coconut milk is influenced by the method used to separate the milk from the coconut. Coconut oil is extracted from the mature fruit’s endosperm and subjected to chemical treatments, such as alkaline treatment and bleaching [31]. VCO is extracted by either supercritical fluid carbon dioxide, enzymatic extraction, centrifugation, fermentation, freezing and thawing method, chilling, low-pressure extraction, hot extraction, and/or cold extraction [32].

Coconut copra has a high quantity of potassium in comparison to other foods [22]. A variety of phytochemicals—including saponins, alkaloids, tannins, glycosides, flavonoids, and phenols—have been discovered in studies on the Nigerian type of coconut. These phytochemicals significantly affect the anti-inflammatory, antioxidant, and reducing properties of the copra [33]. The coconut is protected by a brown, thin outer layer of the endosperm called testa. It is a co-product and by-product of haustorium manufacture and coconut processing industry, respectively. Dehydrated coconut, coconut milk, and virgin coconut oil industry produce testa as a by-product, which is often neglected and utilized as feed for animals. Previously, Appaiah et al. [22] and Marasinghe, Marikkar, Yalegama, Wimalasiri, Seneviratne, Weerasooriya and Liyanage [24] discovered that the testa fraction was an important source of bioactive chemicals, including phenolics and flavonoid contents. Previous research discovered that the lignin content of coconut fiber in the husk and testa was high because of the characterization of the coconut fiber [26,27]. The cellulose content of the coconut fibers was likewise high; however, this could be reduced by the pre-treatment with alkaline solution, NaOH [26].

## 3. Functional Properties

Coconuts provide food, jobs, and business opportunities for millions of people. Because of its intrinsic rich macro and micronutrients profile for human health and nutrition, the fruit is known as ‘wonder fruit’. Commercially processed products include desiccated coconut, oil, raw kernels, milk, and coconut water. Coconut milk and oil production produces coconut cake or flour as a high-fiber, high-protein by-product, created from leftover coconut meal. In coconut meal, glutelin constitutes as the most abundant protein. According to Yalegama et al. [34], dietary fiber can be isolated using the oil meal (poonac) and the coconut residue left over after coconut oil and coconut milk extraction, respectively. It can be utilized as an alternative material for food products, as well as a cheap material for animal feed. Large volumes of unused coconut residue, on the other hand, can be harmful to the environment since it usually ends up rotting. Providing protein and dietary fiber, coconut is included in many food products, for example its flour has been integrated into sweets, snacks, extruded products, and baked goods. It possesses anti-diabetic and anti-cancer properties, as well as the ability to boost immunity and to prevent cardiovascular diseases [35,36]. It is interesting to note that while having a similar nutritional profile to wheat flour, coconut flour is gluten-free. Therefore, a viable and healthful option for the patients of celiac disease includes gluten-free foods utilizing coconut flour.

An ingredient’s functional properties describes its behavior during its preparation and cooking, and its effects to the finished food products with regards to the sensory feel, taste, and appearance. They impact the proteins’ behavior in food in the course of consumption, preparation, storage, and processing [37]. It is crucial to understand the ingredients behavior throughout cooking and preparation, and its effects to the final food product’s flavor, appearance, and texture. Depending on the chemical and physical properties, and the protein size and molecular structure, the functional qualities impact the behavior of food systems throughout consumption, preparation, storage, processing, and manufacturing [38].

Solubility, gelation, water and oil absorption capabilities, and emulsifying and foaming properties are all important functional properties of proteins [39]. Nevertheless, how the protein is extracted, how the derived raw material is processed, including the stages and involved instruments, and the type of raw materials all influence the protein content and functional properties. A study investigating the functional and physicochemical aspects of a protein concentrate derived from a coconut by-product discovered that, at pH 4, coconut proteins were predominantly permeable in highly acidic and alkaline solutions [40]. Extremely alkaline and acidic solutions can solubilize protein powders from coconut cakes. Determining the proteins’ properties will be beneficial in incorporating coconut protein powder into food systems.

### 3.1. Water and Oil Absorption Capacities

The protein powders from coconut oil cake reported lower water and oil absorption capacities than that of coconut milk cake [40]. According to Dat and Phuong [41], for the kernel flour, increasing the flour particles magnitude would reduce the oil absorption capacity; however, it increased the water absorption capacities and swelling.

### 3.2. Oil Holding Capacities

The oil holding capacity is related to the adsorption of organic compounds to the surface of the substrates and an important feature of polysaccharides. While oil holding is partly determined by the chemical composition, it is more determined by the fiber structure’s porosity than by the molecule’s affinity for oil [42,43]. The coconut kernel residues obtained after extracting coconut milk and VCO can provide dietary fibers. Yalegama et al. [34] reported that defatted VCO can be treated chemically to obtain coconut cell wall polysaccharides, exhibiting higher adsorption of oil and oil holding capacity compared to VCO and coconut milk. On the other hand, as opposed to coconut milk, virgin coconut oil has a compact structure with fat, sugar, protein, and minerals. As a result, the oil holding capacity of the virgin coconut oil is reduced by these non-cell-wall components. When compared to virgin coconut oil, coconut milk has more fat but less of the other components. As a result, coconut milk can draw more oil than virgin coconut oil, which has a lower fatty structure.

### 3.3. Protein Solubility

The profile of protein solubility can determine the kinds of beverage or food that can be incorporated with the protein [44]. Coconut protein solubility is normally low—between pH 4 and 5—but it increases if the pH is above or below this range. The range between pH 4 and 5 is the minimum solubility of major protein components from coconut including the extracts from endosperm, coconut skim milk, and coconut protein isolate; this range is also known as the isoelectric point [27,45,46]. The highest solubility was demonstrated at pH 10.3 [45]. When the pH is outside the isoelectric range (pH 3–5), the proteins have net negative or positive charges, causing electrostatic repulsion, ionic hydration, and protein solubilization [46]. Balachandran et al. [47] reported that the solubility of proteins from coconut endosperm also depends on different regions, associated with different amino acid profiles.

### 3.4. Foaming Properties

According to Wani et al. [48], the ability of a protein to generate an interfacial skin to keep air bubbles suspended and prevent them from collapsing when the protein unfolds is called the foaming properties. The denaturation of protein yields more unfolding structures during oil extraction, increasing the interactions at the air–water interface. Furthermore, reducing the surface tension enhances the high foaming capacity, increasing the capacity of a protein to yield good foam. The protein from coconut milk cake has a lower foaming capacity than that from coconut oil cake [49]. pH impacts the foaming capacity of coconut protein isolate. Gonzaliz and Tanchuco [50] showed that the foam expansion was greatest at pH 2 and 11 while the foam stability was low. A thick cohesive layer forms around the air bubble, affecting the foaming stability. Damodaran [49] reported that the foaming stability of protein powders in coconut oil cake and coconut milk cake showed no differences. Wu et al. [38] have reported that peanut and coconut proteins have similar foaming capacity and properties. Foaming properties are important to apply the proteins in food products, such as in whipped toppings, mousses, and beverages.

### 3.5. Emulsifying Properties

According to Senphan and Benjakul [51], proteins are emulsifiers, stabilizing the oil droplets in coconut milk. They stabilize emulsion in two ways: preventing coalescence by generating a cohesive interfacial layer around the oil droplets and reducing the interfacial tension between the water and oil phases. The emulsifying properties of proteinaceous emulsifiers are a measure of their effectiveness based on the emulsifying stability, activity, and capacity [52]. To obtain the emulsifying activity index, the stabilized interface area per unit weight of protein is calculated. Meanwhile, the emulsifying stability index measures an emulsion’s capacity to withstand structural change over a specified time period [53]. The properties are related to the molecular flexibility, surface hydrophobicity, surface charge, and protein solubility. Due to the partial unfolding and dissociation of globular proteins, the hydrophobic groups interact with the denatured proteins, increasing the adsorption at the oil–water interface and surface activity [54]. As a consequence, protein from coconut oil cake had higher emulsifying stability index values and emulsifying activity than that of the protein from coconut milk cake [55].

Proteins in coconut milk affect emulsion stability. A comparative study gauged the emulsion stability and physicochemical parameters of coconut milk derived from three distinct coconut maturation stages [29]. Intrinsic parameters, mainly pH and protein concentration, influenced the stability of coconut milk emulsion. According to Onsaard, et al. [56], proteins extracted from coconut skim milk efficiently stabilized viscous emulsions. However, when compared to whey protein isolate, the proteins extracted from coconut cream had a lesser efficacy due to either the homogenizer’s avoiding droplet aggregation or producing small oil droplets to achieve a stable emulsion [56].

Generally, the coconut proteins’ emulsifying properties are significantly affected by temperature, pH, and ionic strength [17,56]. The emulsion that has 1.2% coconut milk protein and is applied with ultrasound is very stable [57]. Patil and Benjakul [29] investigated the emulsifying properties of fractionated globulin and albumin from defatted coconut meat. When compared to albumin, the globulin fraction performed better as an emulsifier. The coconut proteins showed discrepancies in the emulsifying properties due to amino acid composition changes. The ratios of the protein surface’s polar and nonpolar amino acids, and the changes in the amino acids’ distribution impact emulsifying property. According to the Patil and Benjakul [29], in general, emulsifying properties are higher in hydrophobic proteins with nonpolar side chains. In other words, coconut milk is essentially comprised of emulsion stabilized by proteins in the aqueous phase. Maximizing the emulsifier through protein functionality can improve the coconut milk stability. Nonetheless, destabilizing the emulsion causes its collapse, yielding virgin coconut oil. The procedures used to destabilize coconut milk determine the yield, properties, and qualities of virgin coconut oil.

### 3.6. Thermal Properties

Coconut proteins are very sensitive to heat. Heating them to 80 °C causes denaturation and coagulation [17]. In the high temperature ranging from 80 °C to 120 °C, the raw undiluted coconut milk shows many endothermic transitions through the differential scanning calorimetric measurements. This result reflects the diverse protein composition of coconut proteins as well as their varying heat denaturation behavior [17,58]. The denaturation and precipitation of proteins in coconut milk occur when it is exposed to high temperatures for a long time. Heat enhances the denaturation of coconut protein in both basic and acidic pH regions [56].

## 4. Protein Source

Coconut palm has different parts: leaves, bud, flower, fruit, and germinating nut. Some of these parts are protein-rich, providing benefits for the human health. For example, the protein content in the edible white kernel of the coconut is about 4.3%, mostly consisting of globulin. The essential amino acids present in coconut globulin are shown in Table 1; and these contents are represented as a percentage of the total nitrogen [59]. If the diet for human is complete in other aspects, this is an adequate source of protein, resulting in the same growth even if it is the sole source of protein. Lal et al. [60] reported that feeding proteins isolated from coconut kernel reduces the lipid content in experimental animals because the ratio of lysine to arginine is very low (2.13% lysine and 24.5% arginine). Furthermore, an effective natural coagulant protein is present in the coconut endosperm, and this is a water-soluble protein [61]. As a protein source, copra meal has been fed to ruminants, pigs, and poultry with good results.

Coconut meal is rich in protein (21.2–21.4%, dry matter basis), ideal as a protein source for feeds for livestock [60]. Moreover, in the study by Moorthy and Viswanathan [62], coconut meal contains 22.8% CP, but the critical amino acids in the coconut meal, lysine (0.59%) and methionine (0.34%), are lower than that in other livestock feeds such as soybean meal (2.69 and 0.62%), sunflower meal (1.00 and 0.5%), and groundnut meal (1.54 and 0.54%) respectively, thus it may require supplemental balancing. Although coconut meal contains lower CP and is poor in some essential amino acids (Table 2) compared with other common oil meals, including cocoa by-products and brewer’s grains, it shows higher biological value [63,64]. Moreover, the rumen degradable protein is lower and rumen by-pass protein is higher in coconut meal compared to other feed ingredients [65]. Currently, coconut meal (18–25% CP) is widely used as a protein source for ruminant feeding due to its relatively low cost, but because of its lack of lysine and sulfur amino acids and high fiber content, it is limited for use in non-ruminant diets [66]. Thus, to use it in non-ruminant diets, amino acid supplementation may be required.

Coconut water can also be used to make protein foods by utilizing a culture of the yeast *Saccharomyces fragilis* [60]. However, coconut water has low protein content. Its amino acid composition is shown in Table 2. Similarly, in another part of the coconut palm, the CP in coconut sap (extracted from coconut flower) is 0.26% according to Asghar et al. [67], indicating that coconut sap cannot be considered as a protein source. The properties shown above prove that some parts (e.g., coconut kernel and meal) of the coconut palm are rich with proteins while some (e.g., coconut milk, trunk, and leaves) contain little protein.

## 5. Fiber Source

According to a proximate analysis, a 100 g of coconut flour has 12.1% protein, 10.9% fat, 3.1% ash, 3.6% moisture, and 70.3% carbohydrates [68]. Khan et al. [69] reported that coconut meal contained 6.7% moisture, 1.55% ash, 14.3% protein, 54.0% fat, 20.50% fiber and 23.40% carbohydrates. According to Trinidad et al. [68], while coconut flour contains high levels of dietary fiber, it yields short-chain fatty acids, such as butyrate, acetate, and propionate when fermented. It also has 56.8% insoluble and 3.8% soluble fibers, with a total of 60.9% dietary fiber [70]. Studies also have revealed that coconut flour obtained through dry processing is high in protein whereas flour obtained through wet processing is high in fiber. Coconut flour is a multi-purpose ingredient in developing functional foods with several health benefits [34] as it contain dietary fiber which could be beneficial lowering blood sugar and cholesterol levels, and increasing feces bulk volume. A cheap source of dietary fiber, it can be incorporated in the feed and food industries due to its high in vitro hypoglycemic activity; its effective antioxidant activity against OH, ABTS+, and DPPH radicals; and its high swelling, oil, and water and capacities [71]. According to Adeloye et al. [72], maize flour mixed with defatted coconut flour can potentially decrease protein-energy malnutrition in underdeveloped nations while encouraging the use of coconut residues in food instead of discarding it. Raczyk et al. [73] reported that coconut flour and chestnut flour are both excellent components for both nutritionally enriched and functional foods.

## 6. Antibacterial

According to Boateng et al. [74], coconut oil is an edible oil produced by crushing copra or kernel of matured coconuts collected from the coconut palm [74]. Several studies have investigated the antibacterial activity within the coconut oil. From the fatty acid composition analysis, Hovorková et al. [75] showed coconut oil contained 42% lauric acid, which is a functional medium-chain fatty acid (MCFA) which possesses antiviral and antibacterial activities [76,77]. The MCFA has shown membrane-disruptive activity against Gram-positive bacteria, interfering with bacterial growth or triggering bacterial cell lysis [78]. Hovorková et al. [75] reported that coconut oil was ineffective towards Gram-positive *Bifidobacterium* and *Lactobacillus* spp., but had low antibacterial activities against two tested Gram-negative gut bacteria. However, coconut oil had effectively inhibit *Staphylococcus aureus* at 0.56 mg/mL and *Enterococcus cecorum* at concentration of 1.13–2.25 mg/mL [75]. The *E. cecorum* is recognized as a significant pathogen in broiler, and its infection causes arthritis in chickens [79,80]. Meanwhile, *S. aureus* is observed as a common pathogen that results in systemic disorder in humans and animals [81,82]. Rolinec et al. [83] claimed that coconut oil used as an additive in pig feed could encourage probiotic bacteria to grow, including *Bifidobacterium* and *Lactobacillus* spp., demonstrating a positive effect for pig health management. Coconut meal or oil has been used recently as a feed additive in broiler chickens and pigs to improve their immunity and increase their productivity [83,84,85,86]. Laloučková et al. [87] also revealed a positive antibacterial effect of coconut oil against a pathogenic strain of bovine mastitis-causing bacteria, *Streptococcus agalactiae*. There is promising evidence that using coconut oil that possesses antibacterial properties as a feed additive in animal nutrition could be an alternative solution to replace antibiotics in livestock farming.

In addition, coconut oil constitutes a major product of coconut palm and demonstrates pharmacological properties with potential antibacterial activity against *Streptococcus* spp. That are responsible for tooth decay and extreme gingivitis [88]. Varying in concentration, a coconut alcoholic extract has shown positive results of zone inhibition against *S. mutans*, *Lactobacillus acidophilus*, *S. salivarius*, and *S. mitis* [77]. Lauric acid acts as an antimicrobial compound against some oral cavity contagions. Apart from that, the alcoholic extract of coconut husk fibers has also displayed antibacterial properties against oral dentistry bacteria, such as *Fusobacterium nucleatum*, *Lactobacillus casei*, and *S. mutans* [89,90,91]. The coconut endocarp reported to display a significant antimicrobial effect against *Bacillus subtilis*, *S. aureus*, and *Micrococcus luteus*. However, the extract is not effective on *Escherichia coli* [92]. Another study proved that the choice of solvents used during extraction provided varying antibacterial activities. Benzene extraction of coconut mesocarp powder has shown higher antibacterial activity against *E. coli* while diethyl ether extraction effectively inhibited a pathogenic bacterium, *Salmonella typhi* [93]. The main promising bio-compounds in coconut palm that role in antimicrobial activities are tocopherol, alcohol palmitoleyl, cycloartenol, and β-sitosterol [91]. The previous studies have highlighted many potential antibacterial activities according to the coconut palm composition, and it should be further investigated in different animal models.

## 7. Immunomodulation

Coconut protein’s immunomodulatory characteristics were examined in Swiss albino mice with cyclophosphamide-induced immunological suppression (CP). The CP is a regularly used anti-cancer medication that contains reactive metabolites, such as phosphoramide mustard and acrolein that cause toxicity. According to the findings of this study, coconut protein has a considerable immunomodulatory action [94].

The non-alcoholic palm nectar (NPNC) from *Cocos nucifera* can promote both cell-mediated and humoral immunity according to in vivo data on immune-modulatory activities. In addition, the samples had a hepatoprotective impact, particularly in the presence of increased liver enzymes [95]. The coconut palm’s juvenile inflorescence yields NPNC, which is a bio-refresher and raw material for a variety of value-added goods—including honey, syrup, jaggery, and sugar—made by boiling and thickening the fresh sap [96].

Virgin coconut oil (VCO) contains functional ingredients including 17% meristic acid, 48% lauric acid (LA), 10% capric acid (C10), and 8% caprylic acid (C8) [97,98]. Many recent investigations have found that LA is the most effective bacterial growth inhibitor as the highest zone of inhibition observed on different kind of bacteria [99]. Capric and caprylic acids can also inhibit microbial growth [100]. The VCO has been shown to lessen the growth of *Staphylococcus aureus* by increasing the ability of phagocytic immune cells to destroy bacterial cell walls [9]. It could also be utilized as a cellular immune system modulator and an alternative to antibiotics. Table 3 summarizes the immunomodulatory and medicinal properties of various coconut palm parts from various researchers.

## 8. Antioxidative

Antioxidants in coconut have been reported to be abundant in most plant species, and they can act as reactive oxygen species (ROS) scavengers, improving oxidative stress on sperm function.

Coconut water typically includes 5–8% total soluble solids (TSS), with sugars accounting for the majority (3–7%) of the coconut water. According to Loki and Rajamohan [101], when female rats intoxicated with carbon tetrachloride were provided with 6 mL/100 g body weight coconut water, the antioxidant enzyme activity (superoxide dismutase and catalase levels) was restored and lipid peroxidation was reduced. Coconut water also contains 30 mg/dL L-arginine that inhibits free radical formation due to the antioxidant action [104] and 15 mg/100 mL ascorbic acid that prevents lipid peroxidation in rats [105]. The phenolic components detected in the coconut water were catechin and salicylic acid [106].

In contrast, the chromatographic peaks of eight identified compounds in coconut meat showed that the phenolic compounds in the meat were salicylic, p-coumaric, caffeic, and gallic acids. These chemicals show a significant antioxidant activity in coconut water and flesh. The ascorbic acid levels in coconut meat and water were only 0.9 and 0.7 mg/100 g, respectively [107]. Furthermore, the presence of ascorbic acid was linked to the availability of antioxidant activity in natural coconut water [108].

Coconut testa is a source of various flavonoids and phenolic acids with high antioxidant activity. The synthetic antioxidants can be replaced with these chemicals in food compositions. According to Appaiah et al. [22], there are large amounts of phenolic compounds in the oil produced from coconut kernel containing testa and heat-processed VCO. It is critical to identify the specific polyphenol components and determine the antioxidant capacity in coconut testa to commercialize it as a feed source with antioxidant potential. Compared to commercial coconut oil, VCO has seven times the total phenolic content depending on the types of coconuts and the extraction methods [109] and the refining process may eliminate some of the physiologically beneficial components [110]. A study comparing the antioxidant activity in coconut, goat, and cow milk revealed that coconut has the highest antioxidant activity in total phenol content (TPC), ferric reducing antioxidant power (FRAP), DPPH radical scavenging activity (DPPH), and oxygen radical absorbance capacity (ORAC) assays, with mean values of 575.15 mg GA/100 g FW, 471.55 mg TE/100 g FW, 68.39 percent, and 784.47 umol TE/100g F.W, respectively [111].

## 9. Effects on Ruminants

### 9.1. Methane Production

Several researchers have tried to minimize methane gas emissions in ruminants by feeding cattle feed containing MCFA [112]. Coconut by-products, for example coconut pulp, are used because they contain a lot of MCFA; they are a waste product that is easy to obtain; and they do not compete with human demands [113,114,115]. Besides that, researches have indicated how coconut oil lowers methane gas in rumen fluid fermentation. Sondakh et al. [116] investigated the impacts of dietary VCO as a MCFA source to reduce methane production in the ruminant digestive system. The in vitro experiment included five different VCO treatments using rumen fluid mixed with concentrates and forage substrate in a 40:60 ratio. The following VCO supplementations were used in the experiment, containing increasing levels of VCO (A—0%, B—2%, C—4%, D—6%, and E—8% in dry matter). The study showed that methane production dropped by 18–30% without interfering with the microbial activity of in vitro rumen fluid fermentation following the supplementation of 2–8% VCO in the feed. The addition of up to 4% VCO did not result in a different number of protozoa. However, when the dosage was higher at 6–8% VCO, there was a decrease in protozoa.

### 9.2. Effects on Rumen Fuction

In a different in vitro study, Matsuba et al. [112] investigated the supplementation of 0% and 5% coconut oil in addition to a forage and commercial concentrate at 30:70 ratio. They found that up to 5% oil supplementation had no effect on ruminal total VFA. It raised the concentrations of ammonia and butyrate but reduced the molar percentage of acetate. It also reduced the absolute abundance of genus *F. succinogenes*, with 0.62% for control and 0.002% for coconut oil. Kongmun et al. [117] and Liu et al. [118] observed similar declining trends, indicating lower *F. succinogenes* in vivo and in vitro, respectively. These reductions are the consequence of lauric acid’s bactericidal effect on rumen bacteria [119], which may be species-specific. As a result of this unfavorable effect on *F. succinogenes*, coconut oil may suppress fiber fermentation, which may impede efficient feed utilization.

Few reports demonstrate the impacts of coconut oil on the milk production of dairy cows and dry matter intake (DMI). In this respect, Faciola and Broderick [120] evaluated the dietary supplementation with coconut oil as a practical rumen protozoa-suppressing agent on milk production, nutrient digestibility, ruminal fermentation, and dry matter intake. Coconut oil consumption of 687 g/d in the total mixed ration (TMR) did not affect DMI, milk composition and milk production of lactating dairy cows [120]. Similarly, supplementing 7% coconut oil with garlic powder did not effect on total DMI, ADF digestibility, NDF, and OM in swamp buffalo bulls [117].

Nonetheless, coconut oil’s impact on DMI has been controversial. Hristov et al. [121] put 240 g/d of CO into the rumen of dairy cows before feeding and claimed no change in DMI. However, dairy cows consuming 500 g/d of CO had lower DMI (Lee et al. [122] and no reduction in DMI was observed when beef heifers were fed 250 g/d of coconut oil (Jordan et al. [123]. Reveneau et al. [124] reported that adding 5% coconut oil to the feeds of six lactating dairy cows resulted in a 4.2 kg/d reduction in DMI. When compared to the control diet, Hollmann and Beede [125] reported that substituting coconut oil for ground corn in the feeds for lactating dairy cows reduced DMI and enhanced the ether extract content of the coconut oil diet (10.4% vs. 5.7%, respectively). Furthermore, these authors speculated that the high starch levels (30.1%, DM basis) in the cows’ diets may have increased propionate availability, exacerbating the DMI depression when coconut oil was administered. The unfavorable effects of coconut oil on DMI were documented when coconut oil was used to replace dietary carbohydrates due to a higher fat content in the diet. DMI depression has been linked to the substitution of fats for carbohydrates [126] while possible explanations for the depression include lower NDF digestion, metabolic fuel oxidation, palatability, and gut peptide responses [125].

## 10. Effects on Poultry

### 10.1. Recommended Inclusion Level

Despite having adequate amount of protein and carbohydrate, the usage of coconut meal (CM) in poultry diet is still limited due several factors: insufficient lysine and methionine concentrations, highly indigestible non-starch polysaccharide (NSP), and low bulk density [86]. Earlier works by Eamilao [127] found that broiler chicks tolerated up to 5% CM inclusion in the diets while Mahadevan et al. [128] showed that laying hens nourished with 20% CM were able to maintain their optimum egg production as compared to the corn-soy fed hens. Later, Thomas and Scott [129] observed 40% of CM acceptance by chickens, provided that the diet contained high energy density and was supplemented with lysine. However, the growth was incomparable with the chickens that received a corn-soy diet. The importance of having balanced amino acids (AA) and adequate metabolizable energy (ME) supply in CM diet was later found by Panigrahi et al. [130] and Sundu et al. [131] as they observed that the chickens tolerated up to 25% CM with lysine and methionine supplementation and balanced ME.

### 10.2. Factors Limiting Utilization of Coconut Meal

The presence of NSP in CM including cellulose (13%), galactomannan (61%) and mannan (26%) might be the anti-nutritional factor which may affect the nutrient digestibility in poultry [132,133]. Few studies suggested that the NSP encapsulated the nutrients and hindered digestive enzyme binding to its potential substrate, thus reducing the efficiency of the digestive enzyme hydrolysis [134,135]. Similar works in other NSP-containing feed ingredients have shown depressive effects on the gastrointestinal system and digestive function of chickens, such as mannan in palm kernel cake, pentosan in wheat, and glucan in barley [136,137,138]. Besides that, low lysine concentration was also a main concern when considering CM as poultry feed. With high arginine content, this may in turn lead to an antagonistic effect to lysine availability, meaning that an attention is required when formulating a lysine to arginine ratio [139].

### 10.3. Strategies to Improve Nutritional Qualities of Coconut Meal

Besides amino acid supplementation, other strategies have been explored to improve the feeding values of CM in chicken, including soaking, pelleting, and exogenous enzyme treatment either via pre-feeding fermentation or as a supplementation into the ration. The use of multi-enzyme mixture and mannan degrading enzyme has been found to improve growth rate and reduce mortality in broiler chicken [140,141]. The reduction or degradation of NSP by enzymes increases nutrient availability by breaking down the nutrient-fiber and protein matrix [142]. While soaking or pelleting CM prior to feeding was found to improve the feed intake and growth rate, the nutrient digestibility remained unaffected. Such improvement in feed intake might be due to the changes in CM bulk density, feed solubility, and faster feed passage rate [140].

## 11. Effects on Aquatic Animals

In recent years, many studies investigated the potential application of agricultural wastes including coconut palm in aquaculture sectors [143,144,145,146]. This knowledge is particularly important to solve the major challenge of expensive cost for feed, which is about 70% of the entire feed manufacture. This has the need to identify alternative sources derived from local feed ingredients that are low cost, available, and environmentally friendly. Coconut palm has been reported in many studies to significantly benefit aquaculture species, including digestive, growth, lipid metabolism, health and antioxidative responses [147,148,149,150,151,152]. Even though coconut palm lacks protein because of its low protein and high fiber content, through fermentation technology, the nutrients absorption could be improved [150]. Farizaldi and Jafrinur [151] reported a significant growth of catfish (*Clarias Sp*) fed with fermented coconut waste using bread yeast. Another alternative application of coconut palm is to reduce the feeding cost in aquaculture by using energy-rich oils such as palm oil, coconut oil, fish oil, peanut oil, and virgin coconut oil [153,154,155]. Aside from crude protein and crude fiber, lipid in coconut oil is also evaluated in various studies [146,147,153,156]. Previous studies showed using coconut oil instead of fish oil to improve growth of rainbow trout (*Oncorhynchus mykiss*) [156] and Nile tilapia (*Oreochromis niloticus*) [147,154]. The level of unsaturated fatty acids (EPA and DHA) significantly decreased compared to Herring oil and fish oil [157]. The application of coconut oil is not only limited to fish species, but also on other aquaculture species, such as white shrimp (*Panaeus vannamei*) as reported by [152]. Aside from enhancing growth of aquaculture species, coconut palm showed potential to improve the health of aquaculture species, such as in Nile tilapia (*Oreochromis niloticus*) and *Spinibarbus sinensis* [154,158].

## 12. Conclusions and Future Perspectives

Various parts and products of coconut are being explored and utilized by researchers as an alternative in creating, replacing and reconstructing existing ingredients in animal feed. The presence of rich numbers of various nutrients and beneficial properties turns out to be a major inspiration for the scholars to start delving into coconut. It seems that different coconut products and by-products—such as coconut water, milk, copra, testa, flour, raw kernels, oil, and desiccated coconut—are widely utilized in livestock sectors, especially in ruminant and aquaculture, reporting numerous findings. The discovery is still ongoing. Nevertheless, the occurrence of several anti-nutritional factors such as mannan, galactomannan, cellulose, and others may limit the usage of coconut in the animal feed sectors. Given this obstacle, there is a huge opportunity for research towards technology innovation—such as coconut pre-treatment—in reducing the effects of those anti-nutritional substances before being used as the animal feed while improving the nutritional profiles of the functional parts of coconut. Nonetheless, various significant discoveries are included in this review article, allowing for a somewhat eager outlook of the future. Various parts of coconut can and should be utilized and applied in animal feed to a greater scope. The antioxidant, antibacterial, medicinal, and immunomodulation properties have been discovered in coconut, with all improving the animal health and nutrition. The presence of coconut in animal feed greatly reduces the feeding cost while providing significant benefits to animals, including digestive, growth, lipid metabolism, health, and antioxidative responses and thus offering several potentials to be utilized in the animal feed sector. However, a way of processing, extracting, and treating coconut needs to be established to reduce the negative factors and improve the functional properties of coconut products. Further studies are necessary to enhance the quality of animal products by integrating coconut products into their diets.

## Figures and Tables

**Table 1:** Composition of different coconut parts before and after being applied with different kinds of processing treatments. Data presented on an as-fed basis adapted from previous studies [7,13,14,15,16,17,18,19,20,21,22,23,24,25,26,27].

Coconut Part	Component	Composition	Source
Water	Water	94.18–94.99%	[7,13,14,15,16]
	Protein	0.12–0.72%	
	Total lipid (fat)	0.07–0.2%	
	Ash	0.39–0.87%	
	Carbohydrate	3.71–4.76%	
	Sucrose	0.06–10.7%	
	Glucose	1.48–7.25%	
	Fructose	1.43–5.25%	
	Calcium	27.35 mg/100 g	
	Iron	0.02 mg/100 g	
	Magnesium	6.4 mg/100 g	
	Phosphorus	4.66 mg/100 g	
	Potassium	203.7 mg/100 g	
	Sodium	1.75 mg/100 g	
	Vitamin C	7.41 mg/100 dm^3^	
	Riboflavin (B2)	0.01 mg/100 dm^3^	
Milk	Water	54%	[17,18,19,20,21]
	Moisture	62.6–93.4%	
	Fat	18.83–37%	
	Protein	2–4%	
	Carbohydrates	2–5%	
	Ash	0.63–0.96%	
	Total sugars	0.82–1.62%	
	Total solids	6.6–25.3%	
	Non-fat solids	1.6–2.7%	
	pH	5.9	
Copra	Moisture	3.94–4.3%	[22,23]
	Fat	59.8–71.62%	
	Protein	8.80–10.2%	
	Carbohydrates	6.90–24.3%	
	Crude fiber	7–7.15%	
	Ash	1.4–1.59%	
Testa	Moisture	2.27–4.27%	[22,24]
	Fat	7.93–59%	
	Protein	9.3–32.22%	
	Carbohydrates	26.3–59.24%	
	Crude fiber	11.6%	
	Ash	1.4–5.3%	
Copra oil	Lauric acid	44.84–51.8%	[23,25]
	Myristic acid	18.5–21.86%	
	Caprilic acid	0.13–9.5%	
	Palmitic acid	7.5–9.99%	
	Oleic acid	5.0–8.82%	
	Capric acid	3.5–4.91%	
	Stearic acid	2.71–3.51%	
	Linoleic acid	1.0–1.9%	
Husk	Cellulose	23–55.17%	[26,27]
	Hemicellulose	3–12.26%	
	Lignin	35–45%	
	Ash	0.89–2.56%	

**Table 2:** Amino acid composition of coconut kernel [56] and coconut water [65].

Amino Acids	Coconut Kernel (% of Total Nitrogen)	Coconut Water (% of Total Protein)
Lysine	4.8	1.95–4.57
Threonine	2.7	-
Methionine	1.1	-
Cystine	1.0	0.97–1.17
Tryptophan	0.8	-
Isoleucine	2.9	-
Leucine	4.4	1.95–4.18
Valine	4.1	-
Phenylalanine	2.5	1.23
Arginine	31.0	10.75
Histidine	2.4	1.95–2.05
Alanine	-	2.41
Aspartic acid	-	3.6
Proline	-	1.21–4.12
Serine	-	0.59–0.91
Tyrosine	-	2.83–3.00

**Table 3:** Medicinal properties of various coconut parts, data from previous studies [4,94,101,102,103].

Coconut Parts	Medicinal Properties	Source
Coconut kernel	Antibacterial, antifungal, antiviral, antiparasitic, antidermatophytic, antioxidant, hypoglycemic, hepatoprotective, immunostimulant	[4]
Coconut protein	Immunomodulatory properties	[94]
Coconut water	Hepatoprotective effect	[101]
Virgin coconut oil	Antithrombotic effect	[102]
Coconut oil	Exhibited bactericidal activity against *P. aeruginosa*, *E. coli*, *Proteus vulgaris*, and *Bacillus Subtilis* and has an antiseptic effect	[103]

## Data Availability

All data discussed in this article are publicly available from other sources.
